#  Faecopneumothorax due to missing diaphragmatic hernia: a case report

**DOI:** 10.1186/s13256-020-02606-3

**Published:** 2021-01-23

**Authors:** Resul Nusretoğlu, Yunus Dönder

**Affiliations:** 1 Department of General Surgery, Hakkari Yüksekova State Hospital, Hakkari, Turkey; 2 Department of General Surgery, Kayseri City Training and Research Hospital, Health Science University, Kayseri, Turkey

**Keywords:** Traumatic diaphragmatic hernia, Diaphragmatic injury, Diaphragmatic trauma, Penetrating trauma, Faecopneumothorax

## Abstract

**Background:**

Diaphragmatic hernias may occur as either congenital or acquired. The most important cause of acquired diaphragmatic hernias is trauma, and the trauma can be due to blunt or penetrating injury. Diaphragmatic hernia may rarely be seen after thoracoabdominal trauma.

**Case presentation:**

A 54-year-old Turkish male patient admitted to the emergency department with abdominal pain and dyspnea ongoing for 2 days. He had general abdominal tenderness in all quadrants. He had a history of a stabbing incident in his left subcostal region 3 months ago without any pathological findings in thoracoabdominal computed tomography scan. New thoracoabdominal computed tomography showed a diaphragmatic hernia and fluid in the hernia sac. Due to respiratory distress and general abdominal tenderness, the decision to perform an emergency laparotomy was made. There was a 6 cm defect in the diaphragm. There were also necrotic fluids and stool in the hernia sac in the thorax colon resection, and an anastomosis was performed. The defect in the diaphragm was sutured. The oral regimen was started, and when it was tolerated, the regimen was gradually increased. The patient was discharged on the postoperative 11th day.

**Conclusions:**

Acquired diaphragmatic hernia may be asymptomatic or may present with complications leading to sepsis. In this report, acquired diaphragmatic hernia and associated colonic perforation of a patient with a history of stab wounds was presented.

## Introduction

Diaphragmatic hernias may occur because of a congenital embryonic defect in the diaphragm or can stem from all types of trauma. High-energy blunt or penetrating traumas may cause a rupture in the diaphragm. Penetrating traumas are also divided into gunshots injuries or stab wounds. Diaphragmatic hernias should be considered in patients with a history of blunt or penetrating trauma to the upper abdominal quadrants. The organs such as stomach, small intestine or colon can be incarcerated in the diaphragmatic defect, and ischemia and subsequent necrosis may occur, which may lead to fatal complications. In this case, we present a colon perforation due to a diaphragmatic hernia. The patient had a history of stab wounds three months prior.

## Case presentation

**Fig. 1:**
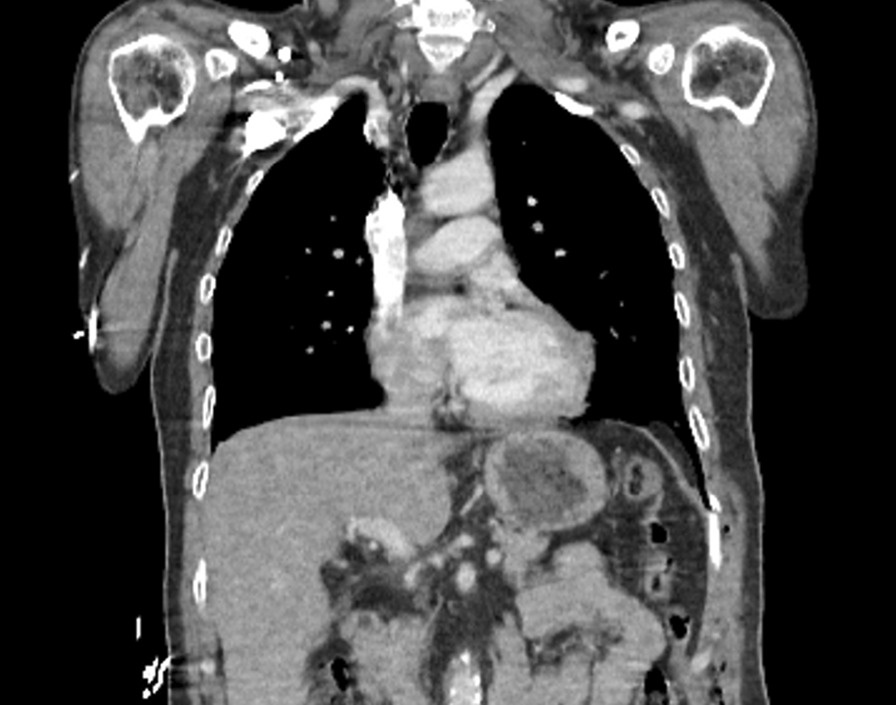
Coronal view of a thoracoabdominal computed tomography scan, after a stab wound 3 months before the diaphragmatic hernia. There is no significant diaphragmatic defect and apparent diaphragmatic hernia

A 54-year-old Turkish male patient admitted to the emergency department with abdominal pain and dyspnea ongoing for 2 days. He had general abdominal tenderness in all quadrants, and lung auscultation revealed decreased respiration sounds on the left. He had a history of a stabbing wound in his left subcostal region 3 months prior without any pathological findings in the thoracoabdominal computed tomography (CT) scan (Fig. 1). The patient was followed up with a daily physical examination. He had no abdominal pain and fever. Oral intake was started. He tolerated the regimen. The patient was discharged on the 3rd day of hospitalization.

**Fig. 2:**
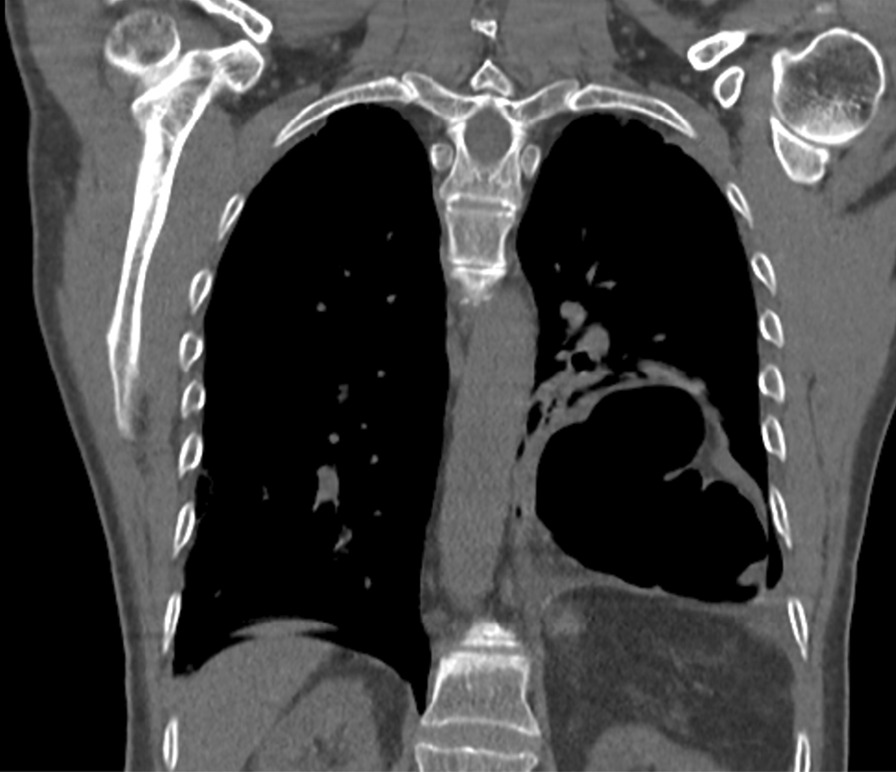
The colon which herniated into the thorax is shown in coronal view of the thoracoabdominal computed tomography scan of the patient, performed at the time of their last admission. (The colon was shown with a red arrow, and fluid was shown with an arrowhead)

**Fig. 3:**
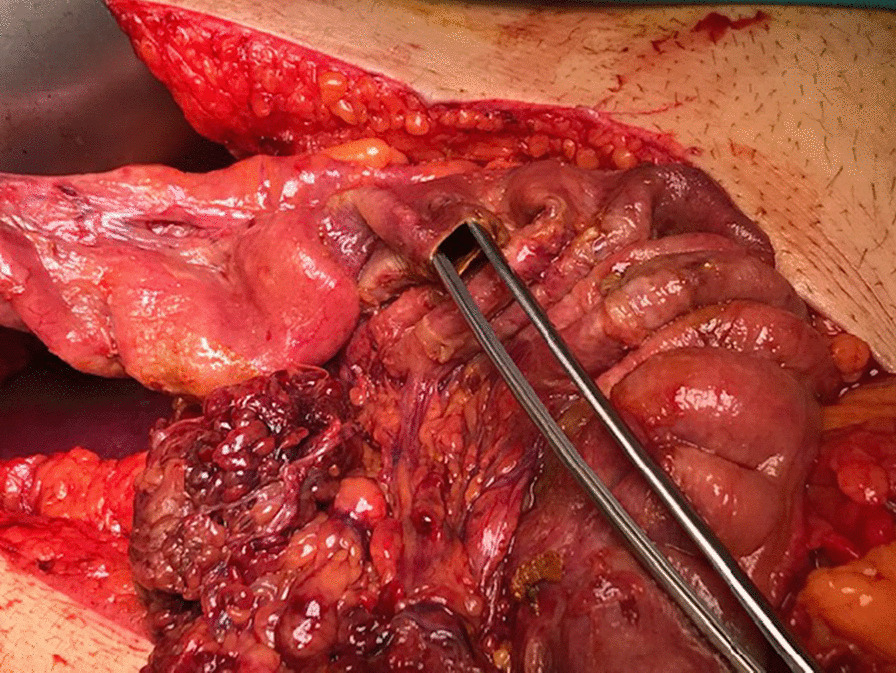
Ischemic colon and site of perforation is shown

**Fig. 4:**
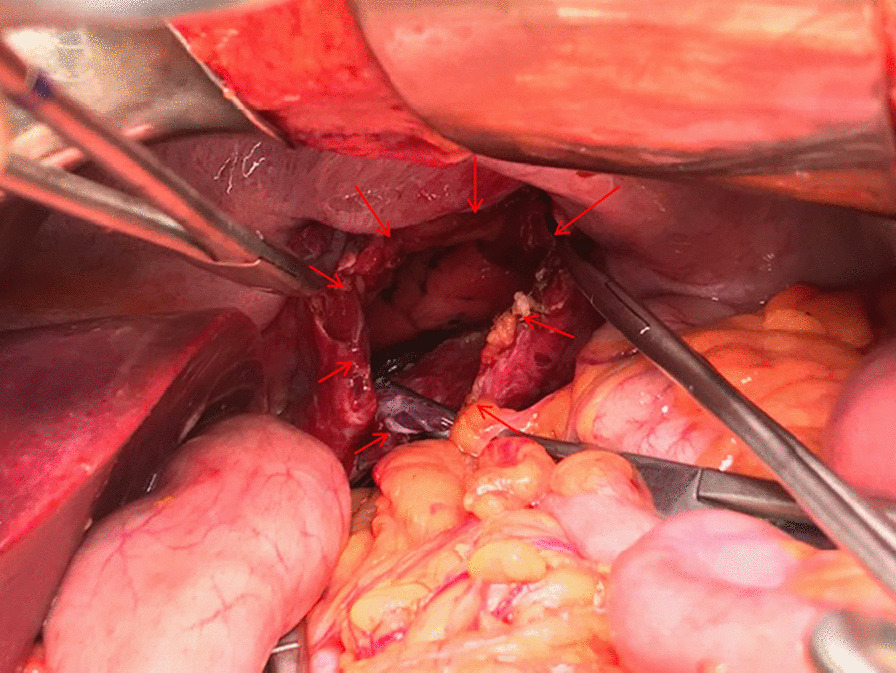
The diaphragmatic defect is shown with arrows

Diaphragmatic hernia was considered due to the history of a stab wound, accompanied by the physical examination findings. Thoracoabdominal CT revealed a diaphragmatic hernia and fluid in the hernia sac (Fig. 2). Due to respiratory distress and general abdominal tenderness, the decision to perform an emergency laparotomy was made. Operative exploration showed the herniation of the transvers colon through the diaphragmatic defect (Fig. 2). After the removal of the dense adhesions, the colon segment was taken into the abdomen. Ischemia, necrosis and perforation were observed in the colon (Fig. 3). There was a 6 cm defect in the diaphragm (Fig. 4). There were also necrotic fluids and stool in the hernia sac in the thorax. The hernia sac and necrotic tissues were excised. It was decided to perform colon resection and anastomosis because the abdomen was clean. The defect in the diaphragm was sutured with a non-absorbable suture. The patient was taken to the intensive care unit for close follow-up. He had respiratory distress. On the 3rd postoperative day, chest X-ray showed fluid accumulation in the basal lobe of the left lung, and thorax tube was performed and exudative fluid was drained. The patient was followed with effective lung care in addition to medical therapy. On the 5th postoperative day, respiratory distress was seen. He was intubated because of acute respiratory distress syndrome (ARDS), which was confirmed with a chest X-ray and decreased saturation. The abdominal drain was taken out on the 6th postoperative day. The patient was started to be fed enterally through a nasogastric tube. ARDS findings regressed during follow-up. The patient was extubated on the 8th postoperative day. On the 9th postoperative day, the nasogastric catheter was removed and oral intake was started. The regime was increased gradually. The thorax tube was taken out. The patient was discharged on the 11th postoperative day.

## Discussion

Diaphragmatic injury is a rare injury occurring in 0.8 to 8% of all traumas [1]. Faecopneumothorax due to colon perforation is a rare condition and may lead to mortality. It is mostly seen secondary to a diaphragmatic hernia on the left side [2]. Diaphragmatic hernias may be congenital or acquired due to trauma. Diaphragmatic traumas are divided into blunt and penetrating trauma, and their incidence is approximately 75%, and 25%, respectively. In penetrating trauma to the lower chest, diaphragm injuries were seen with stab and gunshot wounds (15% and 46%, respectively) [3].

If the laceration of the diaphragm is large, herniation is more easily detected. Sometimes a small laceration in the diaphragm may be asymptomatic in the earlier period after the trauma and therefore cannot be detected. Asymptomatic occult injuries of the diaphragm can turn to a diaphragmatic hernia between 2 months and 50 years after the trauma [4, 5].

A small laceration during trauma can grow over time, causing herniation of organs. Thereafter, herniated stomach, small intestine, omentum or colon can be incarcerated. Patients may present with gastrointestinal symptoms due to herniation or with respiratory problems such as chest pain, dyspnea, tachypnea and cough or hiccups.

Ischemia, necrosis and related perforation can be seen in the organs with hypoxia. It can be a clinical life threatening condition that extends to septic shock.

Sometimes imaging procedures may fail to detect the defect in the diaphragm. Similarly, thoracoabdominal CT of the presented case which was performed for a left upper quadrant stab wound 3 months prior to the admission with abdominal pain and dyspnea did not show any diaphragmatic defect. It is possible that a small laceration increases in size with time and reaches the extent to which the organs can become herniated. Laceration in the diaphragm can be seen in different degrees in blunt traumas, depending on the pressure difference between the abdominal and thoracic cavities. The laceration size also depends on the strength of the diaphragmatic tissue. Diaphragmatic laceration due to blunt trauma usually causes radial tears in the diaphragm, while penetrating injuries are indicated by small holes that follow the size of the projectile [6]. The role of the liver is very crucial, because it prevents bowel herniation from right-sided diaphragmatic injuries [7]. Also, the right side may be slightly stronger. Therefore, a left side diaphragmatic hernia is more common than the right side [3].

A wide variety of symptoms related to diaphragmatic herniation have been described in case reports. In one case, hematemesis was observed due to the bleeding of a herniated gastric fundus. In another case, splenic vein thrombus was observed [8]. Studies indicate that approximately 12–69% of cases are missed preoperatively [8]. In suspected cases, the diagnosis is initially confirmed by chest X-ray, and then tomography, if necessary [9].

If the organs move towards the thorax and decrease the vital capacity of the lung due to herniation in the supine position, the posture of the patient should be adjusted accordingly. Diaphragmatic hernia should be operated. Surgical options are conventional (laparotomy, thoracotomy) or minimally invasive surgery (laparoscopy, thoracoscopy). Mortality rates due to the elective repair of diaphragmatic hernias are low. This rate increases up to 80% in emergency surgery, especially in cases accompanied by necrosis and perforation [10, 11]. Mesh is widely used today in hernia repair. As it is known, the important thing in using mesh is that the surgical area is not infected. We think that mesh should not be used in these cases if possible, since necrosis is seen at a rate of 80% in emergency surgeries in hiatal hernias, and if it is used, it is necessary to choose cases carefully [12].

## Conclusion

Clinicians should be aware of small lacerations in the diaphragm after penetrating abdominal traumas, and could follow the patients for the potential development of diaphragmatic hernias. There may be fatal consequences such as hernia-associated faecopneumothorax. This can be avoided by being suspicious and then maintaining the appropriate approaches.

## Data Availability

Not applicable.

## References

[1] Zarour AM, El-Menyar A, Al-Thani H, Scalea TM, Chiu WC. Presentations and outcomes in patients with traumatic diaphragmatic injury: a 15-year experience. J Trauma Acute Care Surg. 2013;74(6):1392–8. 10.1097/TA.0b013e31828c318e 23694863

[2] Ramdass MJ, Kamal S, Paice A, Andrews B. Traumatic diaphragmatic herniation presenting as a delayed tension faecopneumothorax. Emergency Med J. 2006;23(10):e54. 10.1136/emj.2006.039438 PMC2579619 16988292

[3] Shah R, Sabanathan S, Mearns AJ, Choudhury AK. Traumatic rupture of diaphragm. Annals Thorac Surg. 1995;60(5):1444–9. 10.1016/0003-4975(95)00629-Y 8526655

[4] Lu J, Wang B, Che X, Li X, Qiu G, He S, *et al.* Delayed traumatic diaphragmatic hernia. A case-series report and literature review. Medicine. 2016;95(32):e4362. 10.1097/MD.0000000000004362 PMC4985303 27512848

[5] Singh S, Kalan MMH, Moreyra CE, Buckman RF. Diaphragmatic rupture presenting 50 years after the traumatic event. J Trauma. 2000;49(1):156–9. 10.1097/00005373-200007000-00025 10912874

[6] American College of Surgeons. National Trauma Data Base 2000-2004.

[7] Mallory W, Frankel HL, Turnage R. Recognition and management of diaphragmatic injury in adults. In: Ros BD. eds. Up To Date, Wellesley, MA, 2012.

[8] Wani AM, Al Qurashi T, Rehman SA, *et al.* Massive haematemesis due to strangulated gangrenous gastric herniation as the delayed presentation of post-traumatic diaphragmatic rupture. BMJ Case Rep. 2010;2010:bcr0420102874. 10.1136/bcr.04.2010.2874 PMC3029523 22778192

[9] Ties JS, Peschman JR, Moreno A, Mathiason MA, Kallies KJ, Martin RF, *et al.* Evolution in the management of traumatic diaphragmatic injuries: a multicenter review. J Trauma Acute Care Surg. 2014;76(4):1024–8. 10.1097/TA.0000000000000140 24662867

[10] Christie DB 3rd, Chapman J, Wynne JL, *et al.* Delayed right-sided diaphragmatic rupture and chronic herniation of unusual abdominal contents. J Am Coll Surg. 2007;204:176. 10.1016/j.jamcollsurg.2006.05.007 17189127

[11] Pross M, Manger T, Mirow L, *et al.* Laparoscopic management of a late diagnosed major diaphragmatic rupture. J Laparoendosc Adv Surg Tech A. 2000;10:111–4. 10.1089/lap.2000.10.111 10794216

[12] Yeseob J. Laparoscopic diaphragmatic hernia repair using expanded polytetrafluoroethylene (ePTFE) for delayed traumatic diaphragmatic hernia. Videosurg Other Miniinvasive Techniq. 2017;12:189. 10.5114/wiitm.2017.66373 PMC5502331 28694907

